# Perceived Importance of Abortion Care Features and Access to Telehealth Technologies Among Medication Abortion Patients by Abortion Care Model: Cross-Sectional Analysis of a Prospective Cohort Study

**DOI:** 10.2196/91842

**Published:** 2026-07-15

**Authors:** Rosalyn Schroeder, Antonia Biggs, Finley Baba, Lauren J Ralph, Amy Hagstrom Miller, Colleen McNicholas, Daniel Grossman

**Affiliations:** 1 Advancing New Standards in Reproductive Health (ANSIRH), Department of Obstetrics, Gynecology, and Reproductive Sciences, University of California, San Francisco, 1330 Broadway, Oakland, CA, 94612, United States, 1 502-938-1448; 2 Whole Woman’s Health and Whole Woman’s Health Alliance, Charlottesville, VA, United States; 3 Planned Parenthood Great Northwest, Hawai’i, Alaska, Indiana, Kentucky, St. Louis, MO, United States

**Keywords:** abortion, medication abortion, telehealth abortion, telehealth, patient preferences

## Abstract

**Background:**

Medication abortion accounts for the majority of abortions in the United States, driven in part by the growth in access to telehealth provision of medication abortion. While research indicates high patient satisfaction with telehealth medication abortion care, research on care preferences of patients who use medication abortion remains understudied. Understanding these preferences is essential to informing evidence-based policies that enable people to access person-centered abortion services that meet their values, preferences, and needs.

**Objective:**

The aim of this study is to compare the abortion care features rated as most important among patients receiving medication abortion via telehealth vs in-person care and to assess participants’ access to technologies required for telehealth care. We hypothesized that participants receiving telehealth care would place greater importance on features supporting limited in-person clinic interaction.

**Methods:**

From May 2021 to March 2023, we surveyed participants (≤70 d of gestation, English or Spanish speakers, aged ≥15 y) obtaining medication abortion at 4 organizations providing care in 6 US states. Participants rated the importance of 12 abortion care features (ie, getting care at home, convenience, cost, safety, effectiveness, and privacy) and described access to technologies required for telehealth. We used bivariable logistic and ordinal regressions with robust SEs to assess whether features rated as “extremely important” differed by the medication abortion model received (telehealth vs in-person).

**Results:**

Among 1017 patients approached, 876 were eligible, 583 participants enrolled, 487 initiated a survey, 477 (242 telehealth and 235 in-person) completed survey questions regarding access to technologies for telehealth, and 397 completed questions about abortion care features. Across groups, the features most often rated as “extremely important” included effectiveness (340/397, 85.6%), safety (326/397, 82.1%), timeliness (307/397, 77.3%), and privacy (165/211, 78.2%), with no significant differences (*P*>.05) between groups. Compared to in-person participants, telehealth participants were more likely to report getting care at home (65.9% vs 43.5%; odds ratio [OR] 2.48, 95% CI 1.57‐3.89; *P*<.001) and having a medication abortion (62.1% vs 51.1%; OR 1.58, 95% CI 1.11‐2.23; *P*=.01) as extremely important. They were less likely to report having an in-person meeting with a clinician (16.1% vs 45.7%; OR 0.23, 95% CI 0.19‐0.26; *P*<.001) and ultrasonography (15.2% vs 38.2%; OR 0.28, 95% CI 0.20‐0.40; *P*<.001) as extremely important. Abortion care features rated as extremely important were sometimes discordant with the care received. Almost all participants had access to the technologies required for telehealth.

**Conclusions:**

While telehealth abortion services offer many features that people find important, the availability of both telehealth and in-person abortion care remains critical to ensuring that care aligns with patient preferences. In addition to efforts focused on expanding access to telehealth medication abortion services, advocates and policymakers should continue their work to ensure access to in-person care for those who need or prefer this model.

## Introduction

In 2000, mifepristone, one of the medications in the medication abortion regimen, was approved for use in the United States by the US Food and Drug Administration [1]. Originally, the US Food and Drug Administration required in-person dispensing of mifepristone by a certified clinician, a requirement that was later formalized under a Risk Evaluation and Mitigation Strategy program. In response to the COVID-19 pandemic in 2020, the in-person dispensing requirement for mifepristone was lifted, allowing for mail-order delivery of mifepristone and facilitating the expansion of telehealth medication abortion provision [2]. This shift toward the use of medication abortion accelerated in 2022, when the US Supreme Court removed federal protections for abortion in *Dobbs v. Jackson Women’s Health Organization*, which led to the implementation of total or near-total bans on abortion and resulted in the closure of brick-and-mortar abortion facilities in several states [3]. Following *Dobbs*, abortion shield laws were introduced, providing legal protections for health care providers who offer abortion services to patients living in other states, including those with abortion bans [4].

Together, this constellation of policy changes facilitated the proliferation of telehealth medication abortion, which expanded the ability of health care providers to provide medication abortion outside of the clinic setting [5-7]. In-person medication abortion care typically involves a visit to a facility where patients may receive an ultrasound and/or other screening tests to ensure their eligibility for medication abortion. While the telehealth model may involve a variety of practices, it often eliminates the need for in-person visits and allows patients to access abortion care from their home or other chosen location, with eligibility screening occurring via phone, video, or text, often followed by mail-order delivery of the medication abortion pills [8]. While telehealth medication abortion may involve a variety of practices and facilities may opt to use different models of care [9,10], for the purposes of this paper, we focus primarily on the “no-test” model of telehealth care, which can allow for each component of medication abortion provision to occur without any required in-person visits to a health care facility [8]. We also note that our study was conducted before the implementation of abortion shield laws.

Medication abortion now accounts for the majority of abortions in the United States [11], and telehealth now accounts for 1 in 4 abortions nationally [12,13]. Telehealth abortion has also been implemented in a range of international settings and policy contexts and has been shown to expand access to abortion care [14-18]. A growing body of evidence has found that medication abortion is comparably safe and effective whether provided in-person or via telehealth [10,19,20]. However, it is unclear whether patients choose telehealth or in-person abortion care because it is their preferred model or because other policy or practical constraints push them to use one model over the other. For example, telehealth abortion care is often limited to those eligible for no-test medication abortion (ie, <70 or 77 d of gestation and without other contraindications for medication abortion) and requires access to the internet or other technological devices. This model of care is less available to people living in states with total abortion bans or telehealth abortion bans [7]. Conversely, in-person care may be less private and requires transportation to a facility, which may involve traveling long distances, particularly for those living in rural areas or in states with abortion bans [21,22].

Limited research has examined people’s abortion method and care preferences. Findings from a recent general population survey revealed a preference for medication abortion over procedural abortion and telehealth over in-person care, and found that people tended to prioritize lower-cost care and shorter wait times [23]. While telehealth patients have noted convenience and privacy as important benefits of this model of care [5,24,25], in-person patients have similarly described a preference for privacy and the presence of loved ones during their abortion [26-28]. The extent to which telehealth medication abortion or in-person care models have features that meet patients’ needs and preferences has not been well studied, although it is critical to designing tailored health care approaches that better align with individuals’ values, preferences, and needs.

To address gaps in the literature, this analysis aims to compare the abortion care features that people obtaining medication abortion consider the most important, based on the model of care they received (telehealth vs in-person eligibility assessment). We aim to assess whether participants’ abortion care preferences aligned with the care they received and to examine participants’ access to the technologies required for telehealth abortion care. We hypothesized that participants receiving telehealth care would be more likely to consider features that support limited contact with a facility as extremely important. We report both participants’ ratings of the importance of abortion care features to capture their personal preferences, which may be shaped by external constraints such as abortion policy, eligibility criteria, and the ability to access the model of care (eg, travel, access to technologies, etc).

## Methods

### Recruitment

As part of a prospective, observational study comparing the safety and effectiveness of telehealth versus in-person medication abortion care conducted from May 2021 to March 2023 [19], we recruited participants obtaining medication abortion from 4 abortion-providing organizations in 6 US states, including Colorado, Illinois, Maryland, Minnesota, Virginia, and Washington. These states were selected because they had no restrictions on the use of telehealth medication abortion.

### Recruitment Site Eligibility

To be eligible to participate in the study, partnering organizations must have been providing medication abortion both in-person and via telehealth at the time of participant recruitment. All sites provided synchronous (real-time) care [29], and 2 organizations offered telehealth services via phone or video, while the other 2 offered telehealth only via video.

### Participant Eligibility

Patients were eligible to participate if they were obtaining medication abortion, aged 15 years or older (≥18 y at 4 sites not offering telehealth care to minors), English or Spanish speakers, and ≤70 days of gestation on the date they intended to take mifepristone. Patients younger than the age of 15 years (or <18 y at 4 sites), those who were estimated to be over 70 days of gestation when taking mifepristone, those who were unable to speak English or Spanish, and/or those not seeking medication abortion care were ineligible to participate in the study.

### Data Collection

Approximately 1 day, 2 weeks, and 1 month after receiving their medication abortion pills, we invited participants via email to complete electronic follow-up surveys. For each survey, we sent up to 5 reminders via email, text, or phone, depending on participants’ preferences. Clinic staff abstracted participants’ medical record data approximately 6 weeks after their enrollment.

### Measures

#### Exposures

- *Medication abortion care model received*: this was our primary exposure and included two groups: (1) telehealth (no-test) eligibility assessment by medical history, with either mail or in-person dispensing of medications, and (2) in-person eligibility assessment, with or without ultrasonography. Of note, the primary analysis of this study (published previously [19]) included three study groups: (1) no-test (telehealth) eligibility assessment by medical history and mailing of medications, (2) no-test eligibility assessment (via telehealth or in-person) by medical history and in-person dispensing of medications, and (3) in-person assessment with ultrasonography and in-person dispensing of medications [17]. For this analysis, we recategorized those in group 2 of the original analysis according to whether the eligibility assessment occurred via telehealth or in person (Multimedia Appendix 1).
- *Concordance of abortion care feature preferences with care received*: using medical record data, we created 3 additional secondary binary exposure variables to assess concordance with participants’ ratings of these 3 abortion care features. These included whether: (1) they received ultrasonography (yes/no), (2) they went to a clinic for an in-person visit or in-person dispensing of abortion pills (yes/no), and (3) they met in-person with the clinician providing abortion care (yes/no).

#### Outcomes

- *Perceived importance of abortion care features*: this was our primary outcome, which we collected during the 1-month survey. Our research team developed survey items informed by prior literature [30] and created them in partnership with an advisory board of abortion providers, as well as reproductive justice and health experts. We were guided by an integrated, people-centered health services framework for abortion care, in which the “optimal” model of care is defined by the extent to which care aligns with patients’ values and preferences, rather than being based solely on effectiveness [31]. Using 5-point Likert scale answer options ranging from “not important” to “extremely important,” we asked participants, “Thinking about your *preferences* for having an abortion, please rate how important the following are,” followed by a list of 12 features: the ability to get care at home without a clinic visit, convenience, cost, safety, effectiveness, privacy, meeting in person with a clinician, getting an ultrasound, having a medication abortion versus having a procedure, scheduling the abortion as soon as possible, having as little impact on the participant’s daily life as possible, and the ability to have someone with the participant throughout the abortion (Multimedia Appendix 2 for survey language). To ensure adequate sample sizes, we collapsed the “slightly important” and “somewhat important” categories to create 4-point Likert (extremely important, very important, slightly/somewhat important, and not important) scaled response categories for all analyses.
- A second outcome was *participants’ access to the technologies required for telehealth care* (eg, smartphone, computer, and Wi-Fi) and their experiences with telehealth care, if they received it (measured at the 1-d survey).

At the 1-day survey, we assessed whether participants were offered a telehealth appointment (via phone and/or video), whether participants had a telehealth appointment, and their perceptions of the quality of those appointments, if used. Participant sociodemographic and pregnancy characteristics (age, race or ethnicity, parity, history of abortion, and gestational duration) were also measured at the 1-day survey and were supplemented with medical record data when missing.

### Data Analysis

We describe participants’ sociodemographic characteristics, the perceived importance of each abortion care feature by study group, and participants’ access to technology and experiences with telehealth appointments. To assess differences in participant characteristics by study group, we fit bivariable linear and multinomial regression analyses that accounted for clustering by recruitment site and state, followed by postestimation joint Wald tests assessing the overall association between study group and each variable. To examine differences in the perceived importance of abortion care features, we used bivariable ordinal logistic regression models with 4-point Likert-scale responses (ranging from 1=“not important” to 4=“extremely important”) and logistic regression models for dichotomized outcomes (“extremely important” vs all other responses), with study group as the primary independent variable. To assess whether the care received aligned with participant preferences, we examined 3 abortion care features associated with in-person care (getting an ultrasound; meeting in person with the clinician providing the abortion) or telehealth care (ability to get care at home without a clinic visit) and whether such care was received. To identify potential barriers to accessing telehealth services, we describe patients’ access to technologies and experiences with telehealth appointments. We conducted complete-case analyses for regression models as missing outcome data were low (<3%). All statistical tests accounted for clustering by site and state where abortion care was provided using robust SEs, and analyses were conducted in Stata 18 (StataCorp).

### Ethical Considerations

All study activities were approved by the University of California, San Francisco, Institutional Review Board (20‐32514). Participants provided electronic informed consent to participate in the study. The consent documentation described all measures to maintain patient privacy and confidentiality. We deidentified all survey data prior to analysis, and each participant was assigned a unique study identification number. Participants received a US $15 gift card for enrolling in the study, US $25 gift cards for completing the 1-day and 1-month surveys, and a US $15 gift card for completing the 2-week survey. Medical record data were abstracted, standardized, and entered into a secure REDCap database (Vanderbilt University) by clinic staff.

## Results

### Overview

Among 583 participants enrolled, 487 initiated a survey, and 477 (242 telehealth and 235 in-person) answered at least one of our primary outcome questions and comprise our analytic sample. Among 242 participants in the telehealth group, 63 (26.0%) were dispensed medications in person. Among 235 participants in the in-person group, 35 (14.9%) did not undergo ultrasonography (not shown). Participants in the telehealth and in-person groups differed by age, race or ethnicity, education, and parity; however, both groups had a similar history of previous abortion, receipt of public assistance, and gestational duration (Table 1).

**Table 1.** Demographic and pregnancy characteristics of participants obtaining medication abortions in the United States between May 2021 and March 2023, categorized by study group.

Measure or variable	Overall (N=477), n (%)	Telehealth assessment (n=242), n (%)	In-person assessment (n=235), n (%)	*P* value^a^
Age (y)^b^				<.001^c^
18‐19, n (%)	27 (5.7)	15 (6.2)	12 (5.1)	
20‐24, n (%)	147 (30.8)	59 (24.4)	88 (37.4)	
25‐29, n (%)	147 (30.8)	74 (30.6)	73 (31.1)	
30‐34, n (%)	92 (19.3)	53 (21.9)	39 (16.6)	
≥35, n (%)	64 (13.5)	41 (16.9)	23 (9.8)	
Mean (SD)	27.3 (5.7)	28.2 (6.0)	26.4 (5.5)	.004^c^
Median (IQR)	26 (23‐31)	27 (24‐32)	25 (22‐30)	<.001^c,d^
Race or ethnicity^b^, n (%)				.02^c^
Black (non-Hispanic)	126 (26.4)	54 (22.3)	72 (30.6)	
Hispanic	50 (10.5)	29 (12.0)	21 (8.9)	
Multiracial, Asian, or other	52 (10.9)	21 (8.7)	31 (13.2)	
White (non-Hispanic)	248 (52.0)	138 (57.0)	110 (46.8)	
Missing	1 (0.2)	0 (0.0)	1 (0.4)	
Highest level of education completed, n (%)				<.001^c^
High school or less	127 (26.6)	59 (24.4)	68 (28.9)	
Some college	220 (46.1)	104 (43.0)	116 (49.4)	
Bachelor’s degree or higher	120 (25.2)	73 (30.2)	47 (20.0)	
Missing	10 (2.1)	6 (2.5)	4 (1.7)	
Receipt of public assistance, n (%)				.52
Yes	250 (52.4)	133 (55.0)	117 (49.8)	
No	224 (47.0)	107 (44.2)	117 (49.8)	
Missing	3 (0.6)	2 (0.8)	1 (0.4)	
History of abortion, n (%)				.73
No	275 (57.7)	143 (59.1)	132 (56.2)	
Prior abortion	201 (42.1)	99 (40.9)	102 (43.4)	
Missing	1 (0.2)	0 (0.0)	1 (0.4)	
Parity^b^, n (%)				<.001^c^
0 births	208 (43.6)	91 (37.6)	117 (49.8)	
1‐2 births	192 (40.3)	109 (45.0)	83 (35.3)	
≥3 births	66 (13.8)	37 (15.3)	29 (12.3)	
Missing	11 (2.3)	5 (2.1)	6 (2.6)	
Method of payment for abortion, n (%)				.67
Own or someone else’s money (ie, cash or credit)	264 (55.4)	134 (55.4)	130 (55.3)	
Paid for with help from clinic and/or fund	46 (9.6)	20 (8.3)	26 (11.1)	
Medicaid	100 (21.0)	56 (23.1)	44 (18.7)	
Private insurance or other source(s)	55 (11.5)	26 (10.7)	29 (12.3)	
Missing	12 (2.5)	6 (2.5)	6 (2.6)	
Gestational duration at initial appointment (d)^b,e^				.97
Mean (SD)	46.8 (9.7)	46.8 (10.2)	46.8 (9.0)	

a Following linear or multinomial regression analyses that accounted for clustering by recruitment site and state, *P* values were obtained from postestimation joint Wald tests assessing the overall association between the study group and each variable.

b If age was not reported in the survey data, we filled in the respondent’s age from the patient’s medical record data when available.

c *P*<.05.

d Median differences were assessed using the Mann-Whitney test.

e Gestational duration was based on ultrasonography, and if ultrasonography was not performed, from self-reported date of the last menstrual period.

### Perceived Importance of Various Abortion Care Features

**Figure 1.**
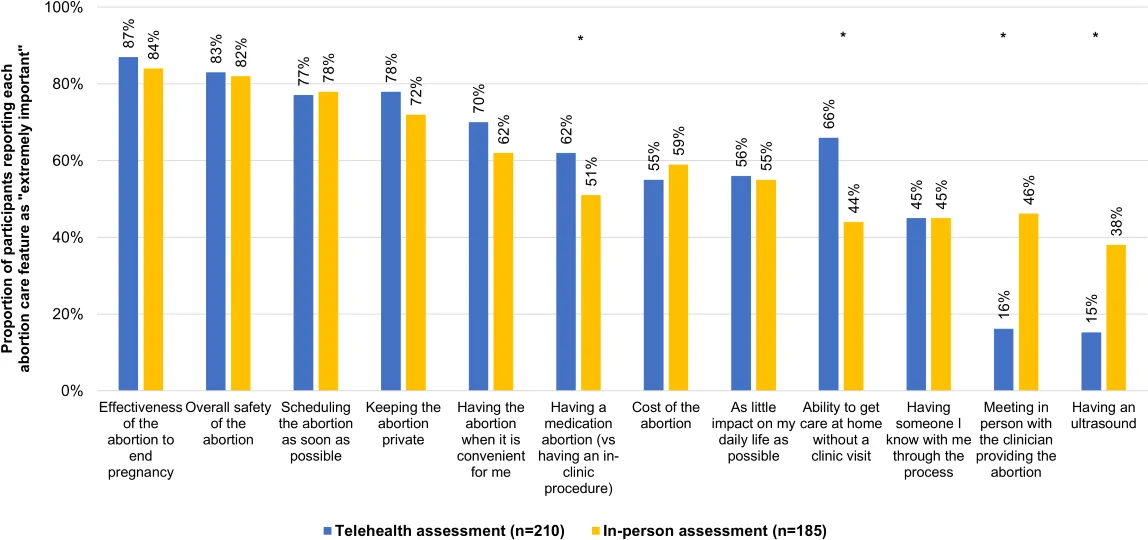
Abortion care features reported as “extremely important” among people obtaining medication abortions in the United States between May 2021 and March 2023, categorized by study group. The symbol “*” indicates *P*<.05 according to logistic regression analyses that account for clustering by recruitment site and the state where abortion care was provided, using robust SEs; effect sizes can be found in Multimedia Appendix 3.

The features most commonly reported as “extremely important” by participants in both study groups included effectiveness (340/397, 85.6%), safety of the abortion (326/397, 82.1%), scheduling their appointment as soon as possible (307/397, 77.3%), and keeping their abortion private (299/397, 75.3%), which did not differ by study group (Table 2 and Figure 1).

**Table 2.** Perceived importance of various abortion care features among participants obtaining medication abortions in the United States between May 2021 and March 2023, categorized by study group.

Abortion care features	Overall (n=397), n (%)	Telehealth assessment (n=211), n (%)	In-person assessment (n=186), n (%)	*P* value^a^
Features associated with telehealth care				
Ability to get care at home without a clinic visit				<.001^b^
Extremely important	220 (55.4)	139 (65.9)	81 (43.5)	
Very important	74 (18.6)	39 (18.5)	35 (18.8)	
Slightly or somewhat important	81 (20.4)	29 (13.7)	52 (28.0)	
Not important	16 (4.0)	2 (0.9)	14 (7.5)	
Missing	6 (1.5)	2 (0.9)	4 (2.2)	
Features associated with in-person care				
Meeting in person with the clinician providing the abortion				<.001^b^
Extremely important	119 (30.0)	34 (16.1)	85 (45.7)	
Very important	57 (14.4)	13 (6.2)	44 (23.7)	
Slightly or somewhat important	117 (29.5)	75 (35.5)	42 (22.6)	
Not important	101 (25.4)	88 (41.7)	13 (7.0)	
Missing	3 (0.8)	1 (0.5)	2 (1.1)	
Having an ultrasound				<.001^b^
Extremely important	103 (25.9)	32 (15.2)	71 (38.2)	
Very important	50 (12.6)	14 (6.6)	36 (19.4)	
Slightly or somewhat important	137 (34.5)	80 (37.9)	57 (30.6)	
Not important	103 (25.9)	84 (39.8)	19 (10.2)	
Missing	4 (1.0)	1 (0.5)	3 (1.6)	
Other features of abortion care				
Effectiveness of the abortion to end pregnancy				.17
Extremely important	340 (85.6)	184 (87.2)	156 (83.9)	
Very important	43 (10.8)	21 (10.0)	22 (11.8)	
Slightly or somewhat important	6 (1.5)	2 (0.9)	4 (2.2)	
Not important	2 (0.5)	1 (0.5)	1 (0.5)	
Missing	6 (1.5)	3 (1.4)	3 (1.6)	
Overall safety of the abortion				.72
Extremely important	326 (82.1)	174 (82.5)	152 (81.7)	
Very important	61 (15.4)	33 (15.6)	28 (15.1)	
Slightly or somewhat important	7 (1.8)	2 (0.9)	5 (2.7)	
Not important	1 (0.3)	1 (0.5)	0 (0)	
Missing	2 (0.5)	1 (0.5)	1 (0.5)	
Scheduling abortion as soon as possible				
Extremely important	307 (77.3)	162 (76.8)	145 (78.0)	.16
Very important	74 (18.6)	42 (19.9)	32 (17.2)	
Slightly or somewhat important	13 (3.3)	6 (2.8)	7 (3.8)	
Not important	0 (0.0)	0 (0.0)	0 (0)	
Missing	3 (0.8)	1 (0.5)	2 (1.1)	
Keeping abortion private				.19
Extremely important	299 (75.3)	165 (78.2)	134 (72.0)	
Very important	53 (13.4)	26 (12.3)	27 (14.5)	
Slightly or somewhat important	33 (8.3)	17 (8.1)	16 (8.6)	
Not important	7 (1.8)	2 (0.9)	5 (2.7)	
Missing	5 (1.3)	1 (0.5)	4 (2.2)	
Having abortion when it is convenient for me				.27
Extremely important	262 (66.0)	147 (69.7)	115 (61.8)	
Very important	89 (22.4)	45 (21.3)	44 (23.7)	
Slightly or somewhat important	38 (9.6)	15 (7.1)	23 (12.4)	
Not important	4 (1.0)	3 (1.4)	1 (0.5)	
Missing	4 (1.0)	1 (0.5)	3 (1.6)	
Having a medication abortion (as opposed to having an in-clinic procedure)				.004^b^
Extremely important	226 (56.9)	131 (62.1)	95 (51.1)	
Very important	80 (20.2)	43 (20.4)	37 (19.9)	
Slightly or somewhat important	76 (19.1)	32 (15.2)	44 (23.7)	
Not important	9 (2.3)	2 (0.9)	7 (3.8)	
Missing	6 (1.5)	3 (1.4)	3 (1.6)	
Cost of abortion				.30
Extremely important	226 (56.9)	116 (55.0)	110 (59.1)	
Very important	78 (19.6)	43 (20.4)	35 (18.8)	
Slightly or somewhat important	76 (19.1)	43 (20.4)	33 (17.7)	
Not important	13 (3.3)	8 (3.8)	5 (2.7)	
Missing	4 (1.0)	1 (0.5)	3 (1.6)	
As little impact on my daily life as possible				.97
Extremely important	220 (55.4)	118 (55.9)	102 (54.8)	
Very important	93 (23.4)	47 (22.3)	46 (24.7)	
Slightly or somewhat important	70 (17.6)	40 (19.0)	30 (16.1)	
Not important	5 (1.3)	2 (0.9)	3 (1.6)	
Missing	9 (2.3)	4 (1.9)	5 (2.7)	
Having someone I know with me through the process				.73
Extremely important	179 (45.1)	95 (45.0)	84 (45.2)	
Very important	66 (16.6)	32 (15.2)	34 (18.3)	
Slightly or somewhat important	95 (23.9)	54 (25.6)	41 (22.0)	
Not important	50 (12.6)	28 (13.3)	22 (11.8)	
Missing	7 (1.8)	2 (0.9)	5 (2.7)	

a *P* values were assessed using ordinal logistic regression and accounted for clustering by recruitment site and state where abortion care was provided, using robust SEs. Odds ratios and effect sizes are presented in Multimedia Appendix 3.

b *P*<.05.

According to logistic regression analyses, telehealth participants were more likely than in-person participants to consider it extremely important to get care at home without a clinic visit (65.9% vs 43.5%; odds ratio [OR] 2.48, 95% CI 1.57‐3.89; *P*<.001) and to have a medication abortion (as opposed to having an in-clinic procedure) (62.1% vs 51.1%; OR 1.58, 95% CI 1.11‐2.23; *P*=.01; Table 2 and Multimedia Appendix 3), and were less likely to consider it extremely important to meet with a clinician in person (16.1% vs 45.7%; OR 0.23, 95% CI 0.19‐0.26; *P*<.001) or get an ultrasound (15.2% vs 38.2%; OR 0.28, 95% CI 0.20‐0.40; *P*<.001).

### Concordance of Ratings of Specific Abortion Care Features With the Care Received

We also assessed whether the care that participants received was concordant with abortion care features they identified as “extremely important.” Among participants who reported that it was extremely important to get an ultrasound, approximately one-third (33/103, 32.0%) did not receive one (Tables 3-5). Among those who thought it was extremely important to meet in person with a clinician, 28.6% (34/119) did not. Among those who felt it was extremely important to get care at home without a clinic visit, 54.1% (119/220) had a clinic visit to meet with a clinician (n=81) or to pick up their medications (n=38). Among those preferring to get care at home but who ultimately had an in-person visit, 56.3% (not shown) lived in a state with a telehealth or total abortion ban in effect.

**Table 3.** Concordance of the rated importance of “having an ultrasound” with the care features received among people obtaining medication abortion in the United States between May 2021 and March 2023 (N=397).

Perceived importance of “having an ultrasound”	Received ultrasonography	Did not receive ultrasonography
Extremely important, n (%)	70 (68.0)	33 (32.0)
Very important, n (%)	31 (62.0)	19 (38.0)
Slightly/somewhat important, n (%)	44 (32.1)	93 (67.9)
Not important, n (%)	13 (12.6)	90 (87.4)
Missing, n (%)	3 (75.0)	1 (25.0)
Total, n (%)	161 (40.6)	236 (59.5)

**Table 4.** Concordance of the rated importance of “meeting in person with the clinician providing the abortion” with the care features received among people obtaining medication abortion in the United States between May 2021 and March 2023 (N=397).

Perceived importance of “meeting in person with the clinician providing the abortion”	Met in person with their clinician	Did not meet in person with their clinician
Extremely important, n (%)	85 (71.4)	34 (28.6)
Very important, n (%)	44 (77.2)	13 (22.8)
Slightly/somewhat important, n (%)	42 (35.9)	75 (64.1)
Not important, n (%)	13 (12.9)	88 (87.13)
Missing, n (%)	2 (66.7)	1 (33.3)
Total, n (%)	186 (46.9)	211 (53.2)

**Table 5.** Concordance of the rated importance of “being able to get care at home without a visit to the clinic” with the care features received among people obtaining medication abortion in the United States between May 2021 and March 2023 (N=397).

Perceived importance of “being able to get care at home without a visit to the clinic”	Received care at home without an in-person visit	Had an in-person visit, including for in-person dispensing of pills
Extremely important, n (%)	101 (45.9)	119 (54.1)
Very important, n (%)	29 (39.2)	45 (60.8)
Slightly/somewhat important, n (%)	25 (30.9)	56 (69.1)
Not important, n (%)	0 (0.0)	16 (100.0)
Missing, n (%)	1 (16.7)	5 (83.3)
Total, n (%)	156 (39.3)	241 (60.7)

### Access to Technology and Telehealth Appointments

Almost all participants in both the telehealth and in-person groups owned a smartphone (99.5% and 96.8%, respectively; OR 6.96, 95% CI 4.24‐11.43; *P*<.001), had access to Wi-Fi (95.6% and 91.7%; OR 1.99, 95% CI 1.12‐3.52; *P*=.02), and most had an unlimited data plan (82.9% and 80.0%; OR 1.14, 95% CI 0.89‐1.47; *P*=.20). Smaller proportions of both groups owned a computer (72.2% vs 73.9%; OR 0.92, 95% CI 0.60‐1.42; *P*=.71) and/or a tablet (55.5% vs 48.4%; OR 1.33, 95% CI 0.82‐2.16; *P*=.25; not shown).

Participants in the telehealth group were significantly more likely than those in the in-person group to report that they were given the option to have a phone appointment (59.9% vs 27.2%; OR 4.08, 95% CI 2.43‐6.85; *P*<.001) and/or a video appointment with a clinician (96.7% vs 23.4%; OR 95.73, 95% CI 50.72‐180.69; *P*<.001) and to have had a phone (50.0% vs 8.5%; OR 10.93, 95% CI 6.24‐19.14; *P*<.001) or video (92.6% vs 1.7%; OR 718.67, 95% CI 95.74‐5394.38; *P*<.001) appointment (Table 6). Among the 34 telehealth participants who reported being offered a phone appointment but declined one, the majority (n=28, 82.4%) preferred a video appointment. Among the 48 participants in the in-person group who reported being offered a telehealth appointment but declined, a majority preferred to be seen in person rather than having a phone (n=37, 77.1%) or video (n=41, 85.4%) appointment.

**Table 6.** Telehealth options offered and reasons for selecting a model of care among participants obtaining medication abortions in the United States between May 2021 and March 2023, categorized by study group.

Measure or variable	Overall (n=477), n (%)	Telehealth assessment (n=242), n (%)	In-person assessment (n=235), n (%)	Odds ratio (95% CI)
Questions about phone appointments				
Offered a phone appointment with clinic or clinician				4.08 (2.43‐6.85)
Yes	209 (43.8)	145 (59.9)	64 (27.2)	
No or not sure	266 (55.8)	95 (39.3)	171 (72.8)	
Missing	2 (0.4)	2 (0.8)	0 (0)	
Had phone appointment with clinic or clinician				10.93 (6.24‐19.14)
Yes	141 (29.6)	121 (50.0)	20 (8.5)	
No or not sure	334 (70.0)	119 (49.2)	215 (91.5)	
Missing	2 (0.4)	2 (0.8)	0 (0.0)	
Main reason participant did not have a phone appointment (n=82)				
Preferred to go in person	38 (46.3)	1 (2.9)	37 (77.1)	—^a^
Preferred to have a video appointment	28 (34.1)	28 (82.4)	0 (0.0)	—
It was easier to go in person	4 (4.9)	0 (0.0)	4 (8.3)	—
Would have had to wait longer for a phone appointment	2 (2.4)	0 (0.0)	2 (4.2)	—
Did not have a phone	1 (1.2)	1 (2.9)	0 (0.0)	—
Worried someone would hear me on phone	1 (1.2)	1 (2.9)	0 (0.0)	—
Other reason (eg, clinic required patient to be in state at time of call, could not get ahold of clinic to schedule phone appointment)	8 (9.8)	3 (8.8)	5 (14.7)	—
Questions about video appointments				
Offered a video appointment with clinic or clinician				95.73 (50.72‐180.69)
Yes	289 (60.6)	234 (96.7)	55 (23.4)	
No or not sure	188 (39.4)	8 (3.3)	180 (76.6)	
Had video appointment with clinic or clinician				718.67 (95.74‐5394.38)
Yes	228 (47.8)	224 (92.6)	4 (1.7)	
No or not sure	249 (52.2)	18 (7.4)	231 (98.3)	
Main reason participant did not have a video appointment (n=55)				
I preferred to go in person	41 (74.5)	0 (0.0)	41 (85.4)	—
I preferred a phone appointment	2 (3.6)	1 (14.3)	1 (2.1)	—
Did not want someone to hear or see appointment	1 (1.8)	1 (14.3)	0 (0.0)	—
Would have had to wait longer for a phone appointment	1 (1.8)	0 (0.0)	1 (2.1)	—
Other reason (eg, poor signal or connection with the provider, and unsure about period timing)	10 (18.2)	5 (71.4)	5 (10.4)	—

a Not applicable.

### Participant Experiences With Telehealth Appointments

Almost all telehealth participants had a video appointment (224/242, 92.6%), and half had a phone appointment (121/242, 50.0%); a small proportion had only a phone appointment (7/242, 2.9%). Telehealth participants largely noted positive experiences with their video- or phone-based care: most participants (213/242, 88.0%) reported feeling very or extremely comfortable asking questions during their appointment, had no difficulty finding a time or place to have their appointment (200/242, 82.6%), and experienced difficulty hearing or seeing staff during their appointment (181/242, 74.8%; Table 7).

Among the 224 participants who had a video appointment, almost all (n=217, 96.9%) reported no difficulty accessing a device to participate in their video appointment. Most participants used a smartphone for their video appointment (n=170, 75.9%), followed by a computer (n=48, 21.4%), a tablet (n=8, 3.6%), or another form of technology (n=3, 1.3%). Over half (52.9%) of those who had a phone or video appointment had it at home, while approximately one-quarter (28.5%) had it in a car or vehicle, among other locations (Table 7).

**Table 7.** Experiences with video or phone appointments among participants obtaining medication abortions via telehealth in the United States between May 2021 and March 2023 (N=242).

Measure or variable	Values, n (%)
Level of comfort asking questions during appointment	
Extremely comfortable	151 (62.4)
Very comfortable	62 (25.6)
Slightly or somewhat comfortable	18 (7.4)
Not comfortable	0 (0.0)
Missing	11 (4.6)
Difficulty finding time or place to have video or phone appointment	
Extremely difficult	0 (0.0)
Very difficult	3 (1.2)
Slightly or somewhat difficult	29 (12.0)
Not difficult	200 (82.6)
Missing	10 (4.1)
Difficulty hearing or seeing staff during video or phone appointment	
Extremely difficult	4 (1.7)
Very difficult	0 (0.0)
Slightly or somewhat difficult	43 (17.8)
Not difficult	181 (74.8)
Missing	14 (5.8)
Location of phone or video appointment (*select all that apply*)^a^	
At home	128 (52.9)
Car	69 (28.5)
At a friend or family member’s house	16 (6.6)
Somewhere else	19 (7.9)
At work	8 (3.3)
Clinic office or parking lot	3 (1.2)
Restaurant or coffee shop	3 (1.2)
Other location	5 (2.1)
Missing	10 (4.1)
Technology used for video appointment among those who had video appointment (n=224; *select all that apply*^a^)	
Smartphone	170 (75.9)
Desktop computer or laptop	48 (21.4)
Tablet	8 (3.6)
Other device	3 (1.3)
Difficulty getting device to have video appointment (n=224)	
Extremely difficult	0 (0.0)
Very difficult	0 (0.0)
Slightly or somewhat difficult	2 (0.9)
Not difficult	217 (96.9)
Missing	5 (2.2)

a Subentries that may sum to greater than the total sample size indicated.

## Discussion

### Principal Findings

In this study of people obtaining telehealth and in-person medication abortion care, participants receiving both models of care reported similar features as extremely important to them: safety, effectiveness, privacy, cost, and scheduling their abortion as soon as possible. These findings are consistent with previous research [23,32]. However, some differences in preferences emerged between the 2 study groups. Compared to the in-person group, telehealth participants were more likely to report that it was extremely important to get care at home without a clinic visit and were less likely to report that getting ultrasonography or meeting with their clinician in person was extremely important. Notably, we found that both telehealth and in-person abortion models of care offer features that are uniquely preferred by many, highlighting that the ability to access both telehealth and facility-based in-person care is critical to addressing patients’ needs and preferences, including those choosing medication abortion.

Still, some participants received a model of care that did not necessarily align with the features they rated as extremely important. While a growing body of evidence supports the provision of medication abortion without ultrasonography [33,34], one-third of participants who noted that having an ultrasound was extremely important to them ultimately did not receive one, highlighting that people’s care preferences are highly individual in nature. Laws that restrict access to abortion may limit abortion patients’ ability to access their desired care preferences and reduce their reproductive autonomy [35]. Additionally, over half of participants who reported that it was extremely important to get care at home without a clinic visit had a clinic visit and/or picked up their medication abortion pills in-person. Regardless of their preferences, some people may have no choice but to visit a clinic if they are ineligible for no-test telehealth abortion care (ie, presenting for care later in pregnancy and/or having a contraindication for medication abortion [8]) or if they live in a state with a telehealth or abortion ban in effect. For example, qualitative interviews with people seeking medication abortion in Mississippi found that many were interested in but unable to access telehealth models of abortion care due to state-level abortion restrictions [36]. However, in the evolving landscape of shield law provision, continued research in this area is needed. It is also important to note that despite some discordance between participants’ abortion care preferences and the care they received, most study participants reported being largely satisfied with the abortion care they received, irrespective of study group [37].

We also found that nearly all participants, including those who received care in person, had access to the technologies required for telehealth abortion. These findings address an important gap in the literature, as many studies have examined the effectiveness and acceptability of telehealth medication abortion care but have not captured abortion patients’ access to the technologies required for this care [38]. Consistent with research showing that people accessing telehealth contraceptive and abortion care are very satisfied with telehealth services [24,39,40], we found that participants who had a phone or video appointment largely noted that it was not difficult to find a device or location, were able to hear or see staff, and were comfortable asking questions during their appointment. These findings suggest that telehealth can provide a feasible alternative to in-person abortion care for those who prefer it, as few people experienced technological barriers to telehealth abortion care. Lastly, while telehealth abortion is often described as facilitating “at-home” abortion care, we found that nearly half of respondents had their phone or video appointment at a location other than their home, which may be an important consideration for designing telehealth abortion care protocols that prioritize patient privacy and autonomy.

### Limitations

This study had some important limitations. Given the observational nature of our study design, participants were not randomized into study groups, and thus their “choice” of model of care received may have been restricted by abortion-related laws in their state of residence, by clinic policies, or by other personal factors that remained unmeasured. While our eligibility criteria included people aged 15 years and older, no youth participants enrolled in our study, limiting the generalizability of our findings to adults. As our recruitment occurred at 4 partner organizations within 6 states, the generalizability of our findings to other contexts also remains limited.

Additionally, participants were asked about their abortion care preferences approximately 1 month after their abortion, and thus their responses may have been influenced by the model of care they received and may not necessarily reflect their preferences prior to obtaining an abortion. Survey items did not undergo rigorous pretesting, although they were informed by prior literature and input from a community advisory board. The survey did not allow participants to rank their care preferences, making it difficult to determine the relative importance of participants’ reported preferences, particularly where features may conflict with one another (eg, where someone reported both a preference for having an ultrasound and for being able to get care at home without a clinic visit).

While we found that almost all survey participants had access to technologies that would allow them to participate in telehealth care, this finding may have been influenced by those who self-selected to participate in the study and/or those who opted to complete subsequent surveys. Additionally, our study was limited to people eligible for medication abortion, thereby excluding the perspectives of people ineligible for medication abortion and/or underrepresenting the views of people who may have preferred procedural abortion. Our sample also excludes those who may have faced insurmountable barriers to accessing abortion care. Furthermore, we conducted the study before states implemented telehealth shield laws, so our findings do not reflect the perspectives of people accessing telehealth abortion under shield laws or through asynchronous care. Future research should explore abortion care preferences among a broader sample of people, including minors, to gain further insight into the factors that matter most to abortion patients.

### Conclusions

This paper offers additional evidence on the care features that patients seeking medication abortion prefer. We found that participants prioritized safety, effectiveness, privacy, and timeliness regardless of the care model they received. Participants receiving telehealth care were more likely to prioritize features that limited in-person interaction, whereas participants receiving in-person care placed greater importance on ultrasonography and in-person clinician interaction. While the abortion care features perceived as extremely important generally aligned with the model of care received, not all participants received the care features they deemed important. This may be, in part, due to a lack of choice, overly restrictive eligibility criteria associated with no-test screening [19,40,41], and/or policy barriers, including telehealth and total abortion bans. Thus, additional research is needed to better understand people’s reasoning for their reported preferences. The variability in the perceived importance of abortion care features across participants also emphasizes the importance of offering a range of medication abortion options. Together, these findings support policies and clinical protocols that allow for autonomous, person-centered approaches where patients can choose their preferred model of abortion care, which would require maintaining access to both in-person care, including optional ultrasonography, and telehealth models of care. We urge health care providers and policymakers to promote evidence-based policies that enable patients to access abortion services that meet their preferences and needs.

## Supplementary material

10.2196/91842 Multimedia Appendix 1 Study group designations.

10.2196/91842 Multimedia Appendix 2 Survey language for abortion care feature preferences question.

10.2196/91842 Multimedia Appendix 3 Odds ratios from logistic and ordinal regressions.

## Abbreviations

OR: odds ratio
