# Supplementary material for: Perceived Importance of Abortion Care Features and Access to Telehealth Technologies Among Medication Abortion Patients by Abortion Care Model: Cross-Sectional Analysis of a Prospective Cohort Study
Source: J Med Internet Res. 2026 Jul 15;28:e91842. doi: 10.2196/91842 (PMC13372261; doi:10.2196/91842)
Supplement: Multimedia Appendix 1 [file jmir-v28-e91842-s001.docx]

**Appendix 1.** Study Group Designations

**Original study categories^1^:**

In-person assessment

(n=235)

Telehealth assessment

(n=242)

In-person assessment with ultrasonography

(n=200)

n=35*

n=200

n=63**

n=179

No-test assessment with pick-up of pills

(n=98)

No-test telehealth assessment with mail-order of pills (n=179)

n=xxx

**Study categories for this analysis:**

* 35 people were assessed for eligibility in person but underwent no ultrasonography/tests.

** 63 people were assessed for eligibility via telehealth but picked up their abortion pills in person.

Notes:

^1^ *Reference:* Ralph LJ, Baba CF, Biggs MA, McNicholas C, Hagstrom Miller A, Grossman D. Comparison of no-test telehealth and in-person medication abortion. JAMA 2024 Sept 17;332(11):898–905. doi: 10.1001/jama.2024.10680.
