# Supplementary material for: Perceived Importance of Abortion Care Features and Access to Telehealth Technologies Among Medication Abortion Patients by Abortion Care Model: Cross-Sectional Analysis of a Prospective Cohort Study
Source: J Med Internet Res. 2026 Jul 15;28:e91842. doi: 10.2196/91842 (PMC13372261; doi:10.2196/91842)
Supplement: Multimedia Appendix 2 [file jmir-v28-e91842-s002.docx]

**Appendix 2.** Survey Language for Abortion Care Feature Preferences Question

*Thinking about your preferences for having an abortion, please rate how important the following are:*

| **Abortion care features** | **Not Important**  (1) | **Slightly Important**  (2) | **Somewhat Important**  (3) | **Very Important**  (4) | **Extremely Important**  (5) |
| --- | --- | --- | --- | --- | --- |
| Overall safety of the abortion | **☐** | **☐** | **☐** | **☐** | **☐** |
| Effectiveness of the abortion in ending the pregnancy | **☐** | **☐** | **☐** | **☐** | **☐** |
| Scheduling the abortion as soon as possible | **☐** | **☐** | **☐** | **☐** | **☐** |
| Little or no travel for the abortion | **☐** | **☐** | **☐** | **☐** | **☐** |
| Meeting in person with the doctor, nurse, or provider providing the abortion | **☐** | **☐** | **☐** | **☐** | **☐** |
| Taking an abortion pill/medication (as opposed to having an in-clinic procedure) | **☐** | **☐** | **☐** | **☐** | **☐** |
| Cost of the abortion | **☐** | **☐** | **☐** | **☐** | **☐** |
| Being able to get my care at home without having to go to the clinic | **☐** | **☐** | **☐** | **☐** | **☐** |
| Having the abortion when it’s convenient for me | **☐** | **☐** | **☐** | **☐** | **☐** |
| Keeping the abortion private | **☐** | **☐** | **☐** | **☐** | **☐** |
| Having an ultrasound | **☐** | **☐** | **☐** | **☐** | **☐** |
| Having someone I know with me through the process | **☐** | **☐** | **☐** | **☐** | **☐** |
| As little impact on my daily life as possible | **☐** | **☐** | **☐** | **☐** | **☐** |
