# Supplementary material for: Perceived Importance of Abortion Care Features and Access to Telehealth Technologies Among Medication Abortion Patients by Abortion Care Model: Cross-Sectional Analysis of a Prospective Cohort Study
Source: J Med Internet Res. 2026 Jul 15;28:e91842. doi: 10.2196/91842 (PMC13372261; doi:10.2196/91842)
Supplement: Multimedia Appendix 3 [file jmir-v28-e91842-s003.docx]

**Appendix 3.** Odds ratios from logistic and ordinal regression analyses examining study group differences in perceiving various abortion care features as “extremely important” among people obtaining medication abortion in the U.S. May 2021-March 2023 (n=397)

| **Abortion care features** | **Logistic regression**  **Odds Ratio (95% CI)** | **Ordinal regression**  **Odds Ratio (95% CI)** |
| --- | --- | --- |
| **Ability to get care at home without a clinic visit** |  |  |
| In-person group (reference) |  |  |
| Telehealth group | **2.48* (1.57, 3.89)** | **2.78* (1.92, 4.00)** |
| **Meeting In-person with the clinician providing the abortion** |  |  |
| In-person group (reference) |  |  |
| Telehealth group | **0.23* (0.19, 0.26)** | **0.15* (0.14, 0.17)** |
| **Having an ultrasound** |  |  |
| In-person group (reference) |  |  |
| Telehealth group | **0.28* (0.20, 0.40)** | **0.21* (0.14,0.30)** |
| **Effectiveness of the abortion to end pregnancy** |  |  |
| In-person group (reference) |  |  |
| Telehealth group | 1.33 (0.89, 1.98) | 1.32 (0.90, 1.92) |
| **Overall safety of the abortion** |  |  |
| In-person group (reference) |  |  |
| Telehealth group | 1.05 (0.74, 1.48) | 1.06 (0.79, 1.45) |
| **Scheduling abortion as soon as possible** |  |  |
| In-person group (reference) |  |  |
| Telehealth group | 0.91 (0.80, 1.03) | 0.96 (0.87, 1.05) |
| **Keeping abortion private** |  |  |
| In-person group (reference) |  |  |
| Telehealth group | 1.31 (0.87, 1.99) | 1.43 (0.87, 2.33) |
| **Having abortion when it's convenient for me** |  |  |
| In-person group (reference) |  |  |
| Telehealth group | 1.38 (0.78, 2.46) | 0.69 (0.37,1.29) |
| **Having a medication abortion (as opposed to having an in-clinic procedure)** |  |  |
| In-person group (reference) |  |  |
| Telehealth group | **1.58* (1.11, 2.23)** | **1.67* (1.12, 2.50)** |
| **Cost of abortion** |  |  |
| In-person group (reference) |  |  |
| Telehealth group | 0.82 (0.56, 1.21) | 0.85 (0.60, 1.23) |
| **As little impact on my daily life as possible** |  |  |
| In-person group (reference) |  |  |
| Telehealth group | 1.03 (0.73, 1.44) | 1.02 (0.75, 1.41) |
| **Having someone I know with me through the process** |  |  |
| In-person group (reference) |  |  |
| Telehealth group | 0.96 (0.57, 1.63) | 1.10 (0.64, 1.92) |

*p<.05. P-values are based on logistic and ordinal regressions that account for clustering by recruitment site and state where abortion care was provided using robust standard errors. In logistic regression, response options were coded as 1=“extremely important” and 0=“very important”, “somewhat important”, or “not important”. For ordinal regression, response options were coded as 4=“extremely important”, 3=“very important”, 2=“somewhat important”, and 1=“not important”.
